# Community-based in situ simulation: bringing simulation to the masses

**DOI:** 10.1186/s41077-019-0112-y

**Published:** 2019-12-21

**Authors:** Barbara M. Walsh, Marc A. Auerbach, Marcie N. Gawel, Linda L. Brown, Bobbi J. Byrne, Aaron Calhoun, on behalf of the INSPIRE ImPACTS investigators, Jessica Katz-Nelson, Khoon-Yen Tay, Travis Whitfill, David Kessler, Robert Dudas, Akira Nishisaki, Vinay Nadkarni, Melinda Hamilton

**Affiliations:** 1 Department of Pediatrics, Division of Emergency Medicine, Boston Medical Center, Boston University School of Medicine, 818 Harrison Ave, Vose 5, Boston, MA 02118 USA; 2 Department of Pediatrics, Yale University School of Medicine, New Haven, USA; 3 Yale New Haven Hospital, New Haven, USA; 4 Department of Pediatrics and Emergency Medicine, Alpert Medical School of Brown University, Providence, USA; 5 Department of Pediatrics, Indiana University School of Medicine, Indianapolis, USA; 6 Department of Pediatrics, University of Louisville School of Medicine, Louisville, USA; 7 Department of Pediatrics and Emergency Medicine, Johns Hopkins University School of Medicine, Baltimore, USA; 8 Department of Pediatrics and Emergency Medicine, University of Pennsylvania Perelman School of Medicine, The Children’s Hospital of Philadelphia, Philadelphia, USA; 9 Department of Pediatrics, Yale University School of Medicine, Yale, USA; 10 Department of Pediatrics, Columbia University Medical Center, Columbia, USA; 11 Department of Pediatrics and Adolescent Medicine, Johns Hopkins University School of Medicine, Baltimore, USA; 12 Department of Anesthesiology and Critical Care Medicine, University of Pennsylvania Perelman School of Medicine, The Children’s Hospital of Philadelphia, Philadelphia, USA; 13 Department of Critical Care Medicine and Pediatrics, Children’s Hospital of Pittsburgh, University of Pittsburgh Medical Center, Pittsburgh, USA

**Keywords:** In situ simulation, Mobile simulation, Emergency department, Quality improvement, Simulation-based medical education, Medical education

## Abstract

Simulation-based methods are regularly used to train inter-professional groups of healthcare providers at academic medical centers (AMC). These techniques are used less frequently in community hospitals. Bringing in-situ simulation (ISS) from AMCs to community sites is an approach that holds promise for addressing this disparity. This type of programming allows academic center faculty to freely share their expertise with community site providers. By creating meaningful partnerships community-based ISS facilitates the communication of best practices, distribution of up to date policies, and education/training. It also provides an opportunity for system testing at the community sites. In this article, we illustrate the process of implementing an outreach ISS program at community sites by presenting four exemplar programs. Using these exemplars as a springboard for discussion, we outline key lessons learned discuss barriers we encountered, and provide a framework that can be used to create similar simulation programs and partnerships. It is our hope that this discussion will serve as a foundation for those wishing to implement community-based, outreach ISS.

## Introduction

Gaps in care exist between academic medical centers (AMCs) and community hospitals, especially regarding the quality of care provided to critically ill neonates and children [1, 2] Only 20% of the 5700 hospitals in the USA are AMC’s and the majority of care is provided in community-based hospital settings [3]. There is an urgent need for creative solutions that enable AMCs to share knowledge and resources with regional and community hospital partners. Such sharing has great potential to assist community hospitals attain increased readiness in neonatal and pediatric acute care medicine, with the ultimate goal of improving pediatric outcomes.

Simulation-based educational methodologies have great potential for enhancing the care provided in community hospitals, but disparities in access to pediatric simulation exist. Many AMC’s have developed robust evidence-based simulation programs to ensure that providers are well-trained and up-to-date on best practices. Community-based hospitals, however, often do not have such programs, and it can be difficult for outside entities to access the resources available at nearby AMC’s [4–11]. To have maximal impact across the entirety of the population, educational modalities such as simulation must be disseminated from academia to community-based hospitals. Fortunately, simulation has developed to the point where AMCs are able to share their resources, curricula, and expertise more readily than in the past [12–18]. In this article, we (1) describe the developments in simulation-based educational methodologies (including mobile simulation, in-situ simulation (ISS), and distributed simulation) that make the dissemination of educational resources outside of AMC’s possible, (2) discuss four examples of community-based ISS programs that have been developed and implemented as collaborations between AMC simulation programs and community hospitals as a way of demonstrating feasibility and value, and (3) provide guidance for those interested in developing similar programs across all healthcare disciplines.

### In situ, mobile, and distributed simulation

In situ simulation (ISS) is defined as simulation that takes place “in the actual patient care setting/environment in an effort to achieve a high level of fidelity and realism.” [19]. Over the past decade, ISS was made possible by the advent of newer, more portable simulation systems, and has allowed simulation-based medical education to more readily be conducted outside of the walls of simulation centers [19, 20]. ISS has also enabled the use of simulation for real-time assessment of care environments [21, 22]. Researchers have explored its utility and versatility, revealing several themes. Kobayashi et al described “portable” simulation, referring to ISS, as a new approach to acute care education and research that added fidelity and realism using the learners’ usual work setting [23]. Several more studies in high risk fields of trauma, pediatric cardiac intensive care, and OB/GYN assessed the use of ISS for interdisciplinary team training, improving team readiness, and enhancing provider comfort during critical events [8, 9, 24–26]. These studies were able to show improvement in simulated patient care that translated into real patient settings after implementation of ISS programs at AMCs [6, 7, 9, 10].

In terms of educational efficacy, ISS stands up well to traditional center-based simulation, as a number of studies have demonstrated equivalent educational outcomes and, in some instances, learner preference for the in situ environment [27, 28]. The use of ISS for detecting latent safety threats and evaluating systems issues (including “readiness” for low frequency events) in the clinical environment has also been assessed via a number of critical studies [14, 26, 29, 30]. It has not only been used to identify knowledge gaps but to reinforce teamwork behaviors as part of regular safety programs [4, 22, 31–34].

ISS programs have been developed and implemented in rural hospitals in the USA and in lower income countries with limited access to simulation centers. Bayouth et al. implemented an ISS curriculum at three rural general emergency departments (ED) and noted that ISS improved provider comfort and performance with pediatric trauma patients [18]. Katznelson et al. similarly implemented a pediatric ISS program at 5 critical access hospitals in North Carolina over a 12-month period, finding that the program was well-accepted and team performance during simulations improved over time [17]. Various types of low fidelity mobile simulation (such as the Helping Babies Breathe program) have also been brought to lower income countries and have been shown to be efficacious [35–38].

In addition to the development of ISS, the technological advances detailed above also allow for easier transport of simulators to external sites, which leads to the concepts of “mobile” and “distributed” simulation. Mobile simulation, “the ability to move the simulator from one teaching location to another or to teach a scenario on the move” involves taking simulation programming “on the road” in a mobile simulation center such as in a recreational vehicle [19]. This new mobility, in turn, results in further possibilities: distributed simulation. Defined as “simulation on-demand, made widely available wherever and whenever it is required,” distributed simulation takes the concept of mobility one step further by focusing on the wide manner in which technology can be made available to learners [19]. Distributed simulation was initially introduced through Kneebone’s description of a transportable mobile operating room environment. This approach has been elaborated on by additional reports describing simulation-adapted recreational vehicles and lower cost mobile simulation centers [10, 13–16, 39–43].

While the definitions above (based on the Society for Simulation in Healthcare dictionary) differ somewhat, the transport of a full simulated environment to external learners is a common thread. This, however, means that the clinical environment of these learners is not used, preventing meaningful assessment of their systems of care. These approaches would thus benefit from the integration of ISS’s focus on systems testing. By combining mobile/distributed simulation with ISS methods, small simulators can be brought from AMC’s to any community hospital’s patient care units. This, in concert with AMC simulation and content expertise, offers the possibility for enhanced community hospital learning as well as on-site environmental safety analysis [22, 25, 44–46]. For simplicity’s sake, we refer to this adapted approach as community-based ISS.

### Exemplar pediatric academic medical center-community hospital programs

In response to the above advancements, our teams developed four community-based ISS that sought to combine mobile, distributed, and in-situ simulation techniques to address disparities in pediatric care in regional community hospitals surrounding ten AMC’s. All four programs were developed in accordance with Kern’s framework of curriculum development [47]. Kern’s construct has six steps which include the following: (1) problem identification, (2) needs assessment for targeted learners, (3) goals and objectives, (4) educational strategies, (5) implementation, and (6) evaluation and feedback. All programs used the Kern’s framework of curriculum development and Table 1 demonstrates how each program adapted and applied the model.

**Table 1:** Program development using Kern’s model of curriculum development

Kern’s steps	Step I/II Def: critical analysis of health care problems and needs assessment and ideal approach	Step III/IV Def: III. Goals and objectives IV. Educational content and educational methods	V. Implementation	Step VI Def: VI. Evaluation and feedback–both learner and program
Norton Children’s KY	Needs assessment at regional transport symposium	Goals and objectives designed by expert review conducted by transport team physicians, nurses, and respiratory therapists Simulation scenarios: non-accidental trauma, septic shock, congenital heart disease	7 institutions engaged—total of 63 participants from different disciplines/professions 3-h sessions with trained simulation faculty Spot debriefing—regular commentary throughout each simulation	Leaner reporting positive feedback with curriculum Endorsed new knowledge acquisition in cognitive, technical, and behavioral skills
Riley Children’s IN	Acknowledgement of deviation from best practice in neonates transferred to the academic center through morbidity and mortality reviews Requests from community providers for delivery room education Needs assessment performed with inter-professional, statewide focus groups	Goals and objectives developed through a multidisciplinary team consisting of neonatal faculty and outreach educators incorporating NRP content	Simulation-based sessions consisting of 30–60 min stations Debriefing with A&I Repetition of the simulations after the debriefing First 2 years: 47 programs and 1300 learners Ongoing programs, approximately 36–48 community hospitals per year	100% learners reported positive learning experience and acquisition of new cognitive, behavioral, and technical skills Multi-professional participants reported increased comfort with range of delivery room procedures Uncovered LSTs involving equipment, medications, resources, personnel, and technical equipment Ongoing research on clinical outcomes impact from the training and from identifying latent safety threats
COMET-MA	Needs assessment based on transfer data to PEDs EDs Acknowledgement of deviation from best practice in patients transferred to the academic center after calling in expects but not implementing management suggested by pediatric emergency attending	Developed goals and objectives designed by a multidisciplinary group of Peds EM attendings and Peds critical care	Initial program 7 participating institutions. Both community EDs and pediatric inpatient units 3 simulations per site Debrief and question and answer session following each simulation case 76 total participants, all multi-disciplinary, MD, PA, RN, RRT, and MAs, all as per their formal code team Ongoing program—any community ER, community health center or EMS service Participants vary by site. Cases are developed to include extended topics including medical cases, trauma and toxicology Programs able to be tailored to site needs	100% of learners reported positive experience. All desired repeat simulation training and elected every 3 months at their site as the best balance for their practice. All levels of participants and disciplines reported increased confidence and comfort in running a code, performing lifesaving procedures in the scope of their practice and had increased medical knowledge in the management of critically ill children Currently given evaluations of system of practice including latent safety threats. Those sites that have repeat visits are being evaluated for change in their system. Polices are being shared such as dextrose dosing, sepsis guidelines, toxicology information sheets. Etc. At community sites are implementing code teams and response teams for pediatric emergency readiness
ImPACTS Northeast Regional Collaborative	Needs assessment based on transfer data to PEDs EDs Feedback from community hospitals on cases of most concern and stress	Larger collaborative developed goals and objectives designed by a multidisciplinary group of ED nurses, Peds EM attendings, Peds critical care, and anesthesia attendings Simulation cases: sepsis, hypoglycemic seizure, FB airway, cardiac arrest	> 200 simulations in the northeast regional collaborative sessions involving over 100 physicians, 300 nurses, and 75 technicians 2.5 h sessions per group, all four cases each followed by standard A&I debrief Standard code team formation per group Recruitment of educational pediatric champion from the community site to partner with AMC	Evaluation of pediatric acute care Systems analysis: med errors, equipment issues, safety assessments Differences in care between high volume and low volume pediatric EDs Site changes–improved relationships between AMCs and community partners Changes in equipment/policies—(HI Flo, protocols)

In each exemplar, portable simulators were used in concert with simulated medications and disposable equipment, developed and refined curricula, and experienced simulation facilitators. Facilities used their own equipment and cognitive aids, except in cases where disposable equipment from the AMC was utilized to allow community hospital exposure to the latest equipment being used at AMCs, or where cognitive aids were specifically developed for use and distribution at the community hospitals.

#### Pediatric acute care transport simulation program, Norton Children’s Hospital/University of Louisville

This program was developed and initiated in 2011 based on information from a need’s assessment conducted at a regional transport symposium. Individual cases and overall course flow were designed by combining this information with an expert review conducted by transport team physicians, nurses, and respiratory therapists. After IRB submission and approval (exemption), the program was piloted at 7 community institutions and reached a total of 63 learners. Participants represented a variety of clinical disciplines. Sessions took approximately 3 h to complete and included three cases per program (abusive head trauma with cerebral edema, meningitis with septic shock, and a new diagnosis of cyanotic congenital heart disease). Case content and complexity was chosen based on the needs assessment and expert review discussed above. Simulations were performed using a concurrent debriefing format, where commentary and debriefing was given at regular intervals throughout the simulation based on the responses of the involved providers [48]. This method was chosen due to the complexity of the cases and the concomitant concern that providers may not be able to navigate them in their entirety without prompting and discussion. All of the participants reported positive experiences with the curriculum and acquisition of new cognitive, technical, and behavioral skills. While the program was initially conceived as an educational pilot, it did evaluate program effectiveness by assessing change in medical knowledge as well as feedback on comfort level in caring for sick pediatric patients. Pre- and post-tests were administered at each session and results indicated improvement in medical knowledge. Testing was based on a score of 100%. Pre-test scores for nurses and physicians were 42% and 60%, respectively, and rose to 64% and 90% post-test following the simulation sessions. Furthermore, other healthcare providers (RRTs, EMTs) also had a clear increase in percentage scored after the simulation sessions. Providers from all disciplines also reported increased comfort with resuscitation of pediatric critical patients following the simulation training. These included overall pediatric critical illness as broken down into respiratory disease, cardiac disease, pediatric advanced life support and procedures during crises. For the majority of participants, the pre-comfort score in most domains on a scale of 1–5 (5 being highest) was 2–2.5 and rose to 4 following the simulation sessions.

#### Neonatal intensive care simulation program, Riley Hospital for Children

This statewide program was established in Indiana in 2011 in response to the identification of neonatal patients receiving less than the standard of care at certain facilities. These facilities were identified through morbidity and mortality reviews of transported neonatal patients in conjunction with community providers’ requests for education. Needs assessments were conducted with focus groups consisting of statewide inter-professional community neonatal providers. Goals and objectives for each case were developed by a multidisciplinary team and were heavily influenced by Neonatal Resuscitation Program content, the gold standard for neonatal resuscitation [49]. Community providers rotated through 3–6 simulation-based stations, each lasting 30–60 min, which consisted of a hands-on simulation followed by facilitated debriefing. Participants were often able to repeat the scenario after the debriefing, allowing for the incorporation of new knowledge and skills. Needs assessment, sample outreach simulation schedules including supply lists, educational strategies, and participant demographics by discipline have been previously published [50]. Identified latent safety threats (LSTs), including those involving equipment, medications, personnel, resources, and technical skills, were reported to each institution with additional follow up 3–6 months later. Table 2 displays LSTs and interventions from 2012 through 2016. In the first 5 years, 47 simulation sessions were conducted, with over 1300 learners at community hospitals participating in the curriculum. One hundred percent of the participants reported a positive learning environment, and acquisition of new cognitive, technical, and behavioral skills. Pre- and post-simulation comfort level data were also collected on routine, moderate, and high-level interventions. Clinicians reported statistically significant improvement in comfort levels across a range of domains, including (a) routine care interventions such as basic neonatal resuscitation, (b) moderate level interventions such assigning APGAR scores and performing bag-mask ventilation, and (c) high level interventions for the resuscitation of critically ill neonates (such as performing endotracheal intubation, placing umbilical catheters, performing chest compressions, and administering medications). Overall comfort improved (*p* values < 0.0001) in 15 of 16 interventions, including the high-level interventions essential for neonatal resuscitation as noted above.

**Table 2:** Riley Children’s Latent Safety Threats—chart of the LSTs in 5 domains at the Riley Children’s NICU outreach program

Community Outreach Latent Safety Threats 2015-2016			
Category	Number of LSTs	Example	Intervention
Equipment	292	Blended oxygen not available in the delivery room. Umbilical catheter kit did not contain catheter, flush or scalpel	Unit created portable blended oxygen set up that is now wheeled to all deliveries. Emergency umbilical catheter kit revised to include necessary items
Medication	18	Teams routinely utilized naloxone for depressed babies during acute resuscitation in the delivery room	Naloxone was removed from newborn delivery room resuscitation medication carts
Personnel	55	A team member is not always present that is designated and able to intubate at deliveries	Additional personnel being trained in intubation
Resource	34	An outdated NRP reference chart is being used for resuscitations Medication chart included outdated dose for epinephrine	New, current NRP algorithms were posted in LDR and nursery Mediation chart revised to include correct dosing of epinephrine
Technical	296	Team members did not trouble shoot ventilation difficulties using MR SOPA prior to initiating chest compressions Teams were unaware of the recommendations to use plastic warp/bag to aid thermoregulation of the extremely premature infant	MR SOPA cognitive tool posted on each warmer to remind staff during newborn resuscitations Premature infant delivery kits were assembled containing plastic bags

The vast majority of issues are technical or equipment related. Examples and interventions implemented by the community site in each LST category are noted in the table

#### Pediatric emergency medicine and emergency medical services simulation

**Fig. 1:**
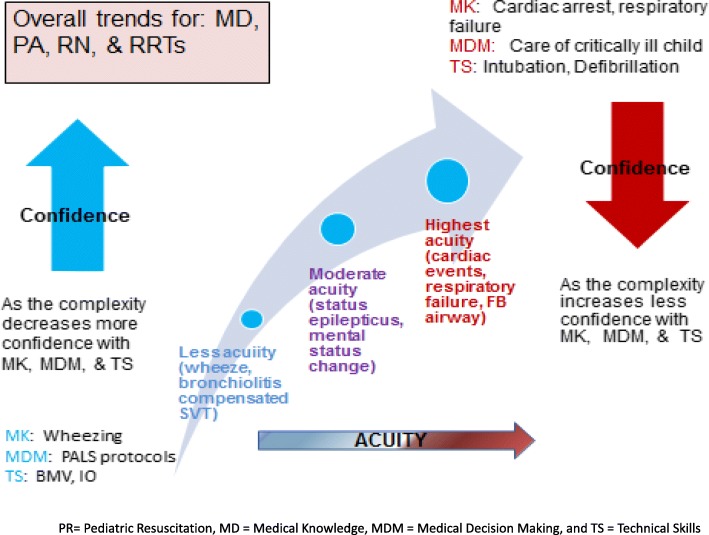
COMET Provider Comfort in Pediatric Emergencies—slide of comfort level in caring for complex critically ill children in community providers

Community Outreach Mobile Education Training (COMET) conducted community-based ISS in the New England region after an initial pilot demonstrated practitioners’ lack of comfort and confidence in caring for acutely ill pediatric patients (Fig. 1). The program was created in response to community hospitals’ desire to continue ISS team training quarterly. COMET uses the community-based ISS model, running multiple pediatric simulation cases in resuscitation bays. COMET has now expanded its focus to include cases on pediatric medicine, toxicology, and trauma, with cases tailored to site-specific needs. A formal assessment of the environment is completed with identification of LSTs, and a feedback document is provided to assist each site in addressing identified areas needing improvement after the ISS. Code cart set-ups have been shared, as have policies and resources addressing specific content from cases, such as evidence-based guidelines and dosing policies. Furthermore, COMET is able to go to community health centers and emergency medical services systems to provide ISS training specific to their units of practice.

#### Pediatric emergency medicine simulation, ImPACTS

Perhaps the most developed and extensive exemplar program is Improving Pediatric Care Through Simulation (ImPACTS). Based on the pilot experience of 2 AMCs, 8 AMCs formed a northeast regional collaborative focusing on community-based ISS. The program’s development pathway included the engagement of community sites through the designation of a partnering “pediatric champion” in each participating general EDs. The ImPACTS curriculum involved four iteratively created scenarios (sepsis, foreign body airway obstruction, cardiac arrest, and seizure) and was implemented in 26 general EDs throughout the northeast USA. The initial needs assessment data revealed that providers of all experience levels were distinctly less confident managing critically ill children, and post-survey data documented community providers’ overwhelming desire to continue simulation activities at regular intervals. In addition to the educational component, this program also offered an assessment of the systems of care at the target institutions, demonstrating that medication errors at these sites were apparent and often multifactorial. To date the program has conducted over 200 simulation sessions involving over 100 physicians, 300 nurses, and 75 technicians. In follow-up discussions with site leaders, there have been changes in processes, equipment set-up, equipment purchasing, and protocol development that are attributable to the program. Stakeholders have reported that this program has improved the relationship between the AMC and community partners during clinical care due to the establishment of ongoing communication with the involved simulation programs. Through this program, disparities in pediatric acute care across a spectrum of EDs have been revealed (Table 3) [51–53].

**Table 3:** Publications from ImPACTS studies and synopsis of data

Author	Year	Topic	Results
Auerbach et al. [13]	2018	Adherence to Pediatric cardiac arrest guidelines	A total of 101 teams from a spectrum of 50 EDs participated. This study demonstrated variable adherence to pediatric cardiac arrest guidelines across a spectrum of EDs. Overall adherence was not associated with ED pediatric volume. Current approaches optimizing the care of children in cardiac arrest in the ED setting are insufficient.
Gangadharan et al. [54]	2018	Inter-personal provider’s perceptions on caring for critically ill infants and children	188 simulation debriefings were recorded in 24 departments, with 15 teams participating from 8 PEDs and 32 teams from 16 GEDs. 24 of the debriefings were transcribed and coded by a multidisciplinary team. Saturation was reached and 4 themes emerged: (1) GED provider comfort with algorithm-based pediatric care and overall comfort with pediatric care in PED, (2) GED provider reliance on cognitive aids versus experience-based recall by PED providers, (3) GED provider discomfort with locating and determining size or dose of pediatric-specific equipment and medications, and (4) PED provider reliance on larger team size and challenges with multitasking during resuscitation. Emerging themes assist in the understanding of provider perceptions.
Walsh et al. [52]	2017	Safety threats during pediatric hypoglycemic seizures	58 teams from 30 hospitals (22 GEDs, 8 PEDs) were enrolled. Pharmacologic based errors occurred more often in GEDs compared to PEDs (*p* = 0.043). Non-pharmacologic errors were uncommon in both groups. Errors with incorrect dextrose concentration occurred more frequent in GEDs (60% vs. 88%; *p* = 0.025), incorrect dose (20% vs. 56%; p = 0.033), and failure to start maintenance dextrose (33% vs. 65%; *p* = 0.040). Overall, PEDs were more likely to select the appropriate concentration and administer the correct dose of glucose.
Auerbach et al. [51]	2016	Differences in pediatric resuscitative care across EDs	58 teams from 30 hospitals participated (22 GEDs, 8 PEDs). This study noted significant differences in the quality of simulated pediatric resuscitative care across a spectrum of EDs. The composite quality score of overall care was higher in PEDs compared with GEDs. The greatest differences in care between GEDs and PEDs were noted for the sepsisand cardiac arrest cases and the teamwork scores.
Kessler et al. [53]	2015	Disparities in adherence to pediatric sepsis guidelines	47 inter-professional teams from 24 EDs. Overall, 21 of the 47 teams adhered to all studied six sepsis metrics (45%). Using standardized in situ scenarios, there was high variability in adherence to the pediatric sepsis guideline across a spectrum of EDs. PEDs demonstrated greater adherence to the guideline than GEDs; however, only composite team experience level of the providers was associated with improved guideline adherence.

Table 3 is a summary of five manuscripts that have been published based on the initial ImPACTS research project, in which the quality of care was evaluated across a spectrum of EDs. There were gaps in care regarding implementation of sepsis guidelines, treatment of hypoglycemic seizures, and adherence to cardiac arrest guidelines. An assessment document was shared with each general ED addressing site-specific gaps and site performance as compared to other general EDs and pediatric EDs. The ImPACTS collaborative has grown and continues to recruit new sites in many more states across the USA.

## Discussion

The above exemplars share the common goal of using community-based ISS methodology to enhance the level of pediatric acute care at community hospital sites. Despite their origination from a number of geographically separated AMC’s and disparate pediatric subspecialty fields, their experiences have been similar in several important ways. In the following discussion, we describe lessons learned, barriers that we have overcome, and a common program development process using Kern’s model that could be used by other AMCs in implementing similar offerings [47].

### Lessons learned

First, each program resulted in the development of new and/or improved relationships between AMCs and the partnering regional community hospital(s). These relationships were instrumental in achieving the educational missions of the community-based ISS programs and have resulted in parallel improvements in clinical interactions between AMCs and community hospitals. Each AMC noted that community providers were more likely to reach out with clinical questions regarding actual patient care after participation in simulation. The relationships have also resulted in clinical collaborations that enable sharing of best practices (evidence-based guidelines for push pull fluids, rule of 50s dextrose dosing sheet, thermoregulation in preterm newborns), policies (family presence during resuscitation), guidelines (sepsis, sickle cell), and reference books/recommended pediatric smart phone apps as well as cognitive aids.

Another aspect of these partnerships is their potential effect on local educational programs. Many of the rural and community hospitals encountered during implementation of these acknowledged having simulation equipment but admitted a complete lack of use due to insufficient expertise. Bringing community-based ISS curricula to these sites thus enabled the creation of ongoing educational partnerships. In fact, the creation of sustainable community simulation-based training programs is being enhanced through the use of simulation instructor courses (SIC) offered at the AMC for community hospital educator partners. This decreases the need for AMC-based facilitators by creating a local core of community educators capable of running the program. The Riley NICU SIC has trained over 50 community partners in the last 3 years. The AMC team continues to partner with these newly trained simulationists to ensure ongoing sustainability.

Another important component of this work is that several of the programs used ISS to assess the environment of care. This feature of ISS is instrumental to quality and safety work, and allowed AMC teams to provide useful feedback to community sites on their systems of care and pediatric preparedness [42]. We recommend that future ISS programs explicitly engage in this type of systems testing as it (1) assists centers in troubleshooting problems of care under the guidance of content experts, (2) generates a collaborative approach toward potential solutions, and (3) allows AMCs to more clearly appreciate the environment of care at community sites.

A further lesson learned is the need for flexibility. Each community hospital’s needs, local context, expectations, and experiences are different, and this must be constantly taken into account when developing and implementing community-based ISS programs. Ultimately, the success of these ISS programs was tied to the ability of each to adapt dynamically to the community institutions they visited. An example of this is the frequent need to deviate from the planned simulation content when necessary based on learner need.

Finally, an organized approach regarding equipment and personnel is needed. Though it may seem intuitive, the equipment must be prepared in a manner that allows easy travel with minimal breakage. It is important to consider set up and breakdown times, consciously designing the program in the most efficient manner possible in order to maximize educational time. One helpful suggestion is to visit the site in advance of the simulation session to assess whether the appropriate equipment is present, as it may be infeasible to use local supplies if present only in limited quantities. We recommend bringing an array of appropriate basic backup equipment.

### A guide for program development

Developing a community-based ISS program is challenging and requires a clear curriculum development framework. To this end, we designed an ignition checklist (Table 4) to organize our main recommendations in a way that we hope will more readily assist those wishing to develop similar programs. Each step in the ignition checklist parallels Kern’s model and has detailed points that should be reflected on as a new program is conceptualized, designed, and implemented [47].

**Table 4:** Ignition checklist for mobile community-based in situ simulation

1. General needs assessment	
□ Connect with outside hospital providers □ Informal discussions with stakeholder clinicians at the putative site regarding needs (bottom–up) □ Formal discussions with administration at putative site regarding needs (top–down) □ Formal discussion with administration at academic medical center regarding felt needs of remote site □ Develop needs assessment questions based on above	
2. Targeted needs assessment	
□ Determine key topics/issues the remote site wants to focus on □ Explore with safety/quality/transport team at academic medical to identify deficiencies in care at site □ Prioritize topic areas □ Identify target learner groups and educators	
3. Goals and objectives	
□ Broad goals: developed optimize patient outcomes □ Define objectives BEFORE case development: specific, measurable, achievable, realistic, timed □ Use objectives to develop cases Construct cases with content experts/inter-professional team (pilot test at your center) Refine cases based on feedback from community □ Pilot cases before site visit to work out kinks, issues—target flow and physiology □ Refinement of cases over time as new or changing needs evolve	
4. Educational strategies/logistics	
□ Establish “no-go” criteria to minimize impact on actual patient flow with community site □ Emphasize need for trauma bay or resuscitation room as adds to realism and can test system □ Plan for best time of day—usually early morning is les busy for EDs □ Plan for travel—equipment, papers, back up technology, power strips, medications, etc. □ Use of unit specific resources (limitations on what can be opened/used) □ Schedule staff members to match	
5. Implementation/sustainability	
□ Sign-up sheets for staff members, schedule far in advance, discuss payment vs. volunteer □ Designate community site champion to get staff excited □ Funding Indirect funding: educational/research grants, non-profit foundation support, donations Direct funding from academic or community medical centers: demonstrate value of program □ Community hospital staff engagement Train the trainer programs Dedicated program liaison personnel (“pediatric/other specialty champion: RN and/or MD”) □ Iterative evolution of academic medical centers role: how much sim, how often	
6. Evaluation and feedback	
□ Evaluations: completed at conclusion of session- computer/paper □ In-person “hot” debriefing—on day of simulation Select format: rapid cycle deliberate practice for psychomotor skills, advocacy/inquiry for complex cases, spot debriefing, after action review model Determine time limit after each case Ensure flow of the session Parking lot—answer other questions through email or after the session Adapt debriefings over time: tele-debriefing, use of video □ Structured systems level debriefing/feedback—within 1 month Academic medical center: on number of transfers, engagement of community/customer Community site with specific action items for improvement Systems integration approach: engagement of quality, safety teams	

The first step in this process involves connecting with a community hospital. While this may seem daunting, the initial connection was well received by most community hospitals in the four described programs. An initial email or phone call followed by an in-person meeting helped to assess interest and potential engagement. Buy-in must be established at the administrative level and the provider level at both the community and AMC site. There are many ways to navigate initial contact and we suggest using existing contacts and partnering with regional entities such as through Emergency Medical Services for Children, American College of Emergency Physician (ACEP) state chapters, and regional perinatal networks of care. Marketing of the program is another area deserving focused attention. For the exemplar programs, such materials were disseminated using mail, regional conference presentations, and the internet (www.bmccomet.com, www.rileychildrens.org/departments/community-outreach-simulation-program, www.impactcollaborative.com). Oftentimes, there is skepticism from participants prior to the session. One way to overcome this barrier is by offering Continuing Medical Education (CME) and Continuing Education Unit (CEU) credits as an incentive. This makes the program more appealing by fulfilling medical certification maintenance requirements.

Curriculum development is crucial to any successful simulation program. Each program found Kern’s framework applicable and effective due to its inherent flexibility [47]. Preliminary program development should focus on balancing the problems felt to be most pressing at the sites of interest with the perceived needs of the supporting AMC where those patients may eventually be transferred. Community sites may recognize gaps or needs, but often have limited resources or challenges in accessing the expertise needed to address them. Additionally, community educators may over- or underestimate the cognitive, technical, or behavioral skills of their teams, creating a barrier to appropriate program design. A specific needs assessment pertinent to each site is thus vital if programs are to have maximum utility. The assessment should focus on inter-professional teams that care for the population in question. Data from this assessment, as well as data obtained from more informal engagement with other potential stakeholders (both from the potential site and the supporting AMC), should be used to inform the development of goals and objectives. To accomplish this, we recommend the establishment of a core group of inter-professional simulation and content experts for curriculum development, implementation, and iterative adaptation over time. Specific content should include best practices as well as scenario features that could potentially reveal deficiencies in patient care and latent safety threats. An interdisciplinary team should trial the cases, refining them using an iterative process to assure that the final product addresses the desired patient physiology, case flow, and learning objectives.

Each program needs to consider a structure and process for debriefing that correspond to the learning objectives. While latitude should be given for unanticipated questions and issues, it is essential for the facilitator to make sure that the key learning points are still communicated. Individual curricula described within this article have used diverse debriefing formats. The decision about debriefing format depends on the experience level of the participants and the relative complexity of the case material, and may need to be made in the moment. Due to this need, we recommend that at least one experienced facilitator/debriefer be present during the initial programmatic offerings, at least until other simulation personnel become fluent with the skills needed to manage this particular educational environment and the variation present in this learner population.

It is critical to have an understanding of each site’s patient flow and to develop preplanned “no go” criteria. Identifying all the equipment and information that will be needed during each visit (monitors, plans for troubleshooting malfunctions, simulated medications, evaluations/surveys, etc.) can be a hurdle, however good communication between faculty can help to ensure smooth programmatic flow. It can also be helpful to visit sites before the educational program if feasible. This allows more accurate planning of space and numbers of participants per session. Unit-specific resources, such as code books, NRP flow diagrams, or department drug reference manual, should also be considered and utilized as much as possible as this significantly contributes to environmental fidelity. All sessions should be concluded with a written evaluation of the program. This enables the educational team to use Kern’s framework to revise and improve the program and, for those programs also focused on systems testing, creates a record of the anticipated changes to be made in the practice or system of care at the local site. Finally, we suggest taking pictures of the room set up and code cart organization as this can enable better feedback to be given on the environment of care as the program proceeds.

Program success depends on having a knowledgeable and dedicated staff. In larger mobile simulation programs, however, carving out protected time for educators to teach and run the simulations can be a barrier to success. Thus, finding means of encouraging this participation, such as training other simulation facilitators to spread the workload at multiple sites, is suggested. Given such a limitation, we further suggest encouraging community site staff involvement with the potential implementation of a “train the trainer” model. Transitioning to this approach will address some of the issues regarding limited protected time for AMC faculty and will ultimately allow AMC faculty to evolve into a consultant role over time.

This naturally leads to a discussion of sustainability strategies. As stated above, offering some form of SICs has proven valuable in our experience. Another critical strategy is to clearly illustrate the program’s value to both the AMC and the community stakeholders as it proceeds. Framing the program as a patient quality and safety initiative is typically more helpful than merely stating it is an educational program.

With regard to ongoing funding, many options exist, including educational grants, research grants, and non-profit foundation support. Local community foundations and donor families can also be an excellent source of funds, especially if the curricula proposed align with their overall mission and interests. Ultimately, partnerships between the AMC and the community sites, with fiscal commitment on both ends, are needed for long-term programmatic viability. For example, the Riley Children’s Hospital NICU program had multiple funding resources that enabled their community-based ISS program to reach sustainability including the Indiana University (IU) Health Values Grant for Education, the Indiana University School of Medicine Neonatal-Perinatal Medicine, and Health/Care Hospital Systems (IU).

Ultimately, sustainability requires the demonstration of value (value = quality/cost) to both the community hospitals and AMC. These programs can enhance value through cost savings for the community site (less risk in patient care), improved quality, and/or increased revenue at AMC’s secondary to additional transfers (patient generated revenue). To continue with the above example, the Riley Children’s Hospital NICU program was able to demonstrate increased referral patterns following outreach simulation programs for two sample community hospitals, and significant spikes in patient referrals were seen after each simulation session for a 1-year period after implementation.

## Future directions

When considering the future of this approach, it is important to acknowledge the current lack of empirical research on the impact of community-based ISS on patient and provider level outcomes. Several recent studies indicate improved outcomes in specific areas of obstetrics and neonatal resuscitation associated with the implementation of center-based and ISS approaches, but these do not address the distributed element of simulation [55, 56]. Most studies consist primarily of curricular descriptions [57]. Hunt’s group, however, was able show improved trauma team performance (provider outcomes) using an ISS intervention in community hospitals [58]. The majority of studies do not report on patient or population level outcomes and more work is needed. The ImPACTS project is currently conducting a project in community sites to describe the impact of the intervention on the outcomes of patients transferred from participating community hospitals. Similarly, the Riley Children’s Hospital NICU program is collecting post session data on improvements in LSTs identified and premature infant admission temperatures.

Finally, the extent to which variants of community-based ISS will impact more traditional modes of simulation-based education must be discussed. At present, AMCs often use simulation centers to cycle large numbers of staff through a standardized educational environment. Outside clinicians participating in such sessions often incur a significant financial cost. Growth of community-based ISS programs, however, have significant potential to alter this balance by providing more accessible, lower-cost, venues at which to obtain training, lessening the need for formal simulation centers in the future. This, in turn, could significantly impact simulation center revenues. Nevertheless, the potential educational, practice, and systems benefits appear to far outweigh this concern. Furthermore, the long-term value of the relationships forged between academic and community sites under this model for both the centers themselves and the patients they serve is immense. Despite the difficulties inherent in embracing new techniques and approaches, we believe this approach represents an optimal means for addressing critical, widespread healthcare issues.

## Conclusion

Healthcare simulation has evolved to the point that community dissemination of ISS programs developed at AMCs is a feasible option. The above program exemplars demonstrate that community-based ISS can be effectively implemented with high acceptance in a variety of clinical settings. Like many fields that have come of age, such as personal computing, the trends toward outward mobility and portability are inevitable, and we encourage programs to consider the possible value that this technique may offer to their community.

## Data Availability

Each site has the data stored on secure servers. It is available upon request.

## Abbreviations

AMC: Academic medical centers
ISS: In situ simulation
ED: Emergency departments
PED: Pediatric emergency department
LST: Latent safety threats
